# Sleep Patterns Across the Transfusion Cycle in Children with Transfusion-Dependent β-Thalassemia: A 14-Day Prospective Controlled Study Using Sleep Diaries and Repeated Questionnaires

**DOI:** 10.3390/children13101339

**Published:** 2026-10-02

**Authors:** Senay Türe, Funda Tayfun Küpesiz, Zeynep Ozturk, Alphan Kupesiz, Elif Guler, Sevtap Velipasaoglu

**Affiliations:** 1 Division of Social Pediatrics, Department of Pediatrics, Akdeniz University Hospital, 07070 Antalya, Turkey; senayture@akdeniz.edu.tr (S.T.); sevtapv@akdeniz.edu.tr (S.V.); 2 Division of Hematology and Oncology, Department of Pediatrics, Akdeniz University Hospital, 07070 Antalya, Turkey; zbolat@akdeniz.edu.tr (Z.O.); akupesiz@akdeniz.edu.tr (A.K.); elifguler@akdeniz.edu.tr (E.G.)

**Keywords:** transfusion-dependent β-thalassemia, sleep diary, sleep quality, sleep variability, sleep regularity, daytime sleepiness

## Abstract

**Highlights:**

**What are the main findings?**

- Sleep diaries indicated greater night-to-night sleep-duration variability and diary-estimated nocturnal fragmentation in children with transfusion-dependent β-thalassemia than in the frequency-matched control group across the 14-day transfusion cycle.
- Questionnaire-reported sleep problems and daytime sleepiness were lower at the post-transfusion assessment, whereas diary-derived sleep-duration variability and nocturnal fragmentation remained elevated; these temporal differences do not establish causality.

**What are the implications of the main findings?**

- Sleep assessment in pediatric thalassemia should extend beyond average sleep duration to include sleep regularity, nocturnal continuity, and daytime functioning.
- Combining multi-day sleep diaries with repeated questionnaires may help distinguish persistent sleep disturbances from symptoms that vary across the transfusion cycle.

**Abstract:**

Background/Objectives: Sleep across the transfusion cycle remains understudied in children with transfusion-dependent β-thalassemia (TDT). We assessed sleep patterns and hematologic correlates before and after transfusion. Methods: This prospective, controlled, repeated-measures study included 27 children with TDT and 30 controls who had been frequency-matched at recruitment on age, sex, and school-attendance category; no fixed one-to-one patient–control pairs were formed. Participants completed 14-day sleep diaries spanning the pre- and post-transfusion weeks and repeated Children’s Sleep Habits Questionnaire (CSHQ) and Pittsburgh Sleep Quality Index (PSQI) assessments. The three primary outcome domains were sleep-diary measures (including sleep regularity, daytime nap frequency, sleep-duration variability, and nocturnal awakenings), continuous CSHQ total and subscale scores, and the continuous modified PSQI global score. Threshold classifications, individual questionnaire items, and laboratory/MRI associations were exploratory. Multiplicity was controlled within the diary and questionnaire families using the Benjamini–Hochberg false discovery rate (FDR). Results: Within the primary sleep-diary domain, weekday sleep irregularity was greater in patients pre-transfusion (16.3 vs. 10.2 min; q = 0.014) but not post-transfusion (q = 0.220); sleep-duration variability was greater before and after transfusion (both q = 0.026), and diaries recorded more daytime naps and more and longer nocturnal awakenings across 14 days (q < 0.05). Within the primary questionnaire domains, the continuous modified PSQI global score and CSHQ total, sleep-behavior, and daytime-sleepiness scores were higher in patients pre-transfusion (all q < 0.001). In exploratory, unadjusted analyses, lower pre-transfusion hemoglobin correlated with greater daytime sleepiness (ρ = −0.425; *p* = 0.027). Conclusions: Multiplicity-adjusted findings across the three primary outcome domains suggest differences in reported sleep timing, regularity, continuity, daytime napping, and questionnaire scores in children with TDT. However, the small sample, the number of component outcomes and contrasts, and the absence of actigraphy require cautious interpretation.

## 1. Introduction

Transfusion-dependent β-thalassemia (TDT) is a severe hemoglobinopathy characterized by ineffective erythropoiesis and chronic anemia, requiring regular erythrocyte transfusions from early childhood throughout life. Regular transfusions, effective iron chelation, and monitoring of organ iron overload via magnetic resonance imaging have resulted in significant gains in growth, organ preservation, and survival. The current Thalassemia International Federation guidelines recommend transfusions every 2–5 weeks in most TDT patients, with pre-transfusion hemoglobin levels maintained in the range of 9.5–10.5 g/dL [1,2]. However, contemporary care should aim not only to monitor hemoglobin and iron levels but also to maintain the child’s daily functioning and health-related quality of life.

Despite improved survival, the burden of TDT extends beyond chronic anemia and organ complications. Repeated hospital visits, transfusion schedules, chelation therapy, and fatigue can affect family routines, school attendance, and peer participation. Sleep supports growth, neurodevelopment, attention, learning, emotional regulation, and academic functioning [3], while disrupted sleep is associated with daytime sleepiness, irritability, impaired concentration, and lower quality of life. Sleep problems may therefore add to the developmental and functional burden of TDT but remain overlooked in routine hematologic care.

Sleep health cannot be defined solely by average sleep duration; timing, nocturnal integrity, day-to-day variability, regularity, and daytime function are complementary dimensions. The within-individual standard deviation of total sleep duration indicates sleep variability, while the within-individual standard deviation of sleep midtime indicates timing irregularity. These measures can reveal recurring short and long nights, or a mismatch between biological time and social time created by school and family schedules, even if the average duration appears normal [4,5]. Tissue hypoxia, fatigue, and decreased daytime activity due to chronic anemia, considered alongside the role of iron in myelination and neurotransmitter metabolism, suggest a plausible biological relationship between hemoglobin levels and sleep–wake patterns.

In contrast, the sleep literature is limited in children with transfusion-dependent thalassemia. A small polysomnography study reported sleep fragmentation, periodic limb movements, and objective daytime sleepiness in children and adolescents with β-thalassemia [6]. In Güneş’s cross-sectional study comparing 76 children with β-thalassemia major to 68 healthy controls, the total score on the Children’s Sleep Habits Questionnaire, as well as scores for nocturnal awakenings and sleep-related breathing disturbances, were higher in the patients [7]. Studies evaluating different transfusion-dependent hemoglobinopathies together have shown a similar burden of problems, but mixed diagnostic groups have limited thalassemia-specific inferences [8]. Poor subjective sleep quality, prolonged sleep latency, insomnia, and fatigue have been reported in adult or wide-age thalassemia samples; however, associations with ferritin and hemoglobin have not been consistent [9,10].

Much of the available evidence comes from cross-sectional surveys or single-night observations, which cannot characterize night-to-night instability or short-term variation across repeated transfusion cycles. Pediatric studies following the same patients with multi-day diaries and repeated pre- and post-transfusion questionnaires, while including concurrent healthy controls and measures of sleep variability and mid-sleep timing, remain scarce. It is therefore unclear whether differences in subjective sleep quality observed at the post-transfusion assessment co-occur with differences in nocturnal integrity and sleep-timing regularity.

This evidence gap is relevant to balancing hemoglobin targets and iron burden in TDT management. Serum ferritin alone does not fully represent organ iron; organ imaging, individualized chelation, and adherence remain central to monitoring [1,2]. Although higher pre-transfusion hemoglobin has been associated with better pediatric health-related quality of life [11], such observations do not establish that lower hemoglobin or a longer transfusion interval causes sleepiness or impaired functioning. Sleep and daytime function should therefore be considered complementary outcomes in future transfusion-strategy studies.

Therefore, this study aimed to characterize sleep patterns in children aged 3–18 years with transfusion-dependent thalassemia using sleep diaries and questionnaires administered at defined pre- and post-transfusion time points. We examined sleep quality, daytime functioning, sleep variability, and sleep-timing regularity and explored their associations with hematologic and iron-related measures [7,12,13].

## 2. Methods

### 2.1. Research Design

This prospective, observational, well-controlled, repeated-measures study was conducted between 15 March and 1 June 2025, in collaboration between the Thalassemia Center and the Social Pediatrics Department of the Pediatric Hematology Division at Akdeniz University Faculty of Medicine. The study evaluated sleep patterns in transfusion-dependent thalassemia patients aged 3–18 years throughout a transfusion cycle using sleep diaries and sleep questionnaires.

### 2.2. Setting and Sample of the Study

Sample size was determined a priori using two complementary calculations that informed two components of the primary outcome framework. First, based on the Pittsburgh Sleep Quality Index (PSQI) scores reported for the patient and control groups by Şener et al. (5.57 ± 3.07 and 3.38 ± 1.82, respectively), we calculated the standardized effect size as Cohen’s d = 0.89 [9]. A two-sided independent-samples test conducted in G*Power version 3.1.9.4 (Heinrich Heine University Düsseldorf, Düsseldorf, Germany), with α = 0.05, 90% power, and equal group allocation, indicated that at least 27 participants per group (54 in total) were required for the continuous PSQI global score. Second, we performed a complementary calculation for diary-derived sleep regularity using the approach described by Fischer et al. [14]. Assuming group-specific day-to-day variability in mid-sleep time of 30 and 60 min, a total of 27 participants were required to achieve 80% power for the between-group comparison; after allowing for a 15% attrition rate and maintaining balanced allocation, the diary-based target was set at 34 participants (17 per group). Accordingly, we planned to enroll 60 participants (30 patients and 30 controls) to accommodate potential attrition and incomplete data. The study was not separately powered for every component measure within the sleep-diary and CSHQ primary domains or for exploratory threshold, item-level, and laboratory/MRI analyses; these results were therefore interpreted using multiplicity-adjusted q values, effect estimates, confidence intervals, and consistency across periods.

#### Study Population and Eligibility Criteria

The age limits were prespecified before recruitment. Forty-five children with transfusion-dependent β-thalassemia were followed at the study center. Children younger than 3 years were excluded a priori because sleep at this developmental stage is more strongly characterized by daytime napping and because the study questionnaires were not extended below the lower pediatric age supported by the literature. Thirty-eight patients were aged 3–18 years; of these, 34 met the remaining eligibility criteria and were invited to participate. The upper age limit corresponded to the pediatric scope of the center.

Children were eligible for the patient group if they were 3–18 years of age, had a diagnosis of transfusion-dependent β-thalassemia major, were receiving regular transfusions, and agreed to complete the sleep diaries and questionnaires. Patients were excluded if they had transfusion-independent thalassemia or did not adhere to the regular transfusion program; were outside the prespecified age range; had a comorbid psychiatric disorder; used medication that could affect sleep; reported tobacco, alcohol, or other substance use; or did not complete the required sleep-diary or questionnaire assessments. Pre-transfusion hemoglobin values were not used to determine eligibility, and no minimum or maximum hemoglobin threshold was applied when selecting participants.

Controls were recruited from children aged 3–18 years who attended the Social Pediatrics/Healthy Child Outpatient Clinic for routine preventive follow-up. Eligibility was determined before administration of the study instruments, based on routine clinical assessment and review of the child’s medical history. Eligible controls had no known chronic or psychiatric disorder, no physician-diagnosed sleep disorder, no current medication use for a sleep-related complaint, and no regular medication use. At recruitment, controls were selected by group-level frequency matching to the enrolled patient group on age, sex, and school-attendance category. Matching was not performed as fixed one-to-one patient–control pairs, and no individual control was linked analytically to a specific patient. The CSHQ, modified PSQI, sleep diary, and all other study outcome measures were not used to determine eligibility or exclude potential controls; they were administered only after enrollment. We invited 34 eligible patients; four families declined participation, and we enrolled 30 patients and 30 frequency-matched controls. Three patients subsequently withdrew because they did not wish to continue the sleep diary. No controls were removed or replaced and no post-withdrawal rematching was performed; therefore, the final analyses included 27 patients and 30 controls as two independent groups.

### 2.3. Data Collection and Measurement Tools

Both groups kept sleep diaries for 16 consecutive days. For each enrolled patient, monitoring was prospectively aligned with the next routinely scheduled transfusion cycle and was not initiated or timed according to an observed pre-transfusion hemoglobin result. The transfusion day marked the end of the patient’s routine intertransfusion interval, when hemoglobin was expected to be relatively lowest within that cycle; the pre-transfusion hemoglobin value was measured as part of routine clinical care on that day and was not used to select the participant or study days. The diary was intended to characterize sleep across the intertransfusion cycle rather than sleep on a single low-hemoglobin day. The 16-day recording window comprised seven analyzable days before transfusion and seven analyzable days after transfusion, together with the hospital admission/transfusion day and the following day. The latter two days were prespecified for exclusion in both groups because mandatory early waking and acute disruption of daily routines could distort habitual sleep; therefore, 14 days were analyzed. Control-group monitoring was scheduled over corresponding calendar periods. Results were calculated for the pre-transfusion, post-transfusion, and overall 14-day periods and were summarized by weekdays, weekends, and all days.

Questionnaires were administered in person on the transfusion day before transfusion and by telephone 8–10 days after transfusion. At each assessment, participants were instructed to report on the preceding seven days rather than on the day of questionnaire administration alone. Control-group assessments were conducted over corresponding periods. The PSQI and CSHQ were completed with the parent and child present together. Both informants’ accounts were considered when each item response was recorded. For children aged 3–10 years, parental reporting contributed more substantially because of developmental differences in comprehension and recall, while the child’s account was incorporated whenever feasible. Separate parent- and child-specific scores were not obtained. Sociodemographic and clinical data, as well as current hemoglobin before and after transfusion, annual mean pre-transfusion hemoglobin, ferritin, and organ iron load MRI results, were obtained from the most recent available records. Thus, administration mode was systematically linked to assessment time and was neither randomized nor counterbalanced.

#### 2.3.1. Children’s Sleep Habits Questionnaire (CSHQ)

The Children’s Sleep Habits Questionnaire (CSHQ) is a 33-item parent-reported scale developed by Owens et al. to assess children’s sleep habits and sleep problems [15]. Fiş et al. conducted a Turkish validity and reliability study; they reported a Cronbach’s alpha of 0.78 and a test–retest correlation of 0.81 [16]. The Cronbach’s alpha coefficient in this study was 0.76.

The scale consists of four main sub-dimensions: bedtime resistance, sleep behavioral problems (parasomnias, sleep-related breathing disorders), nocturnal awakenings, and daytime sleepiness. Items are scored using a three-point Likert scale, and total scores of 41 and above are considered clinically significant sleep problems. In this study, participants were asked to answer the scale questions considering the previous 7-day period.

#### 2.3.2. Pittsburgh Sleep Quality Index (PSQI)

The original Pittsburgh Sleep Quality Index (PSQI) is a self-reported instrument that assesses sleep quality over the preceding month [17]. The Turkish version was validated by Ağargün et al. [18]. It comprises seven components—subjective sleep quality, sleep latency, sleep duration, habitual sleep efficiency, sleep disturbance, sleep medication use, and daytime dysfunction—each scored from 0 to 3, yielding a global score from 0 to 21. To align questionnaire assessment with each seven-day sleep-diary period, we used a study-specific 7-day modified PSQI: the original items, components, and scoring algorithm were retained, but the reference period was changed from one month to the preceding seven days. Pediatric psychometric evidence includes an Italian validation study in healthy children aged 3–16 years [19], and abbreviated PSQI reporting periods have been used in previous studies [20,21,22,23,24,25]. At both assessments, the PSQI was administered using the joint parent–child procedure described above; therefore, the resulting score represents a multi-informant assessment rather than uniform child self-report or exclusive parent-proxy report. The Cronbach’s alpha coefficient in the present study was 0.78. The global score was analyzed primarily as a continuous variable. For secondary exploratory analyses, we applied a threshold of ≥5. This threshold has pediatric support in a validation study of the Chinese PSQI among childhood cancer survivors aged 6–18 years, in which ROC analysis against DSM-5 insomnia criteria identified ≥5 as the optimal integer cut-off (AUC = 0.83, sensitivity = 0.81, specificity = 0.70) [23]. Because that study involved a different language, clinical population, standard one-month recall period, and self-report procedure, ≥5 was not interpreted as a validated clinical cut-off for the modified administration used here.

#### 2.3.3. Sleep Diary and Calculation of Sleep Diary Parameters

In this study, we used a consensus-based standard sleep diary [26]. Table 1 summarizes the definitions of the parameters evaluated in the sleep diary analysis. The calculations were based on two example studies by Fisher et al. and Morales-Ghinaglia et al. [14,27].

Sleep diary parameters were calculated for the pre-transfusion, post-transfusion, and the entire 14-day period, separated into weekdays, weekends, and all-week (weekday + weekend). The day of hospital admission and the following day were excluded from the calculations. When evaluating the diaries, mean sleep duration, variability in mean sleep duration, the midpoint of mean sleep duration, and sleep regularity were considered as separate measures. This approach revealed alternating short and long nights and instability in sleep timing, even when mean duration (hours) was similar. Variability and regularity were calculated for the pre-transfusion, post-transfusion, and the entire 14-day period, separated into weekdays and all-week (weekday + weekend). A higher standard deviation indicated more variable duration and lower timing regularity, respectively. Weekend sleep variability and regularity could not be calculated because at least 3 days of data were needed to calculate the standard deviation.

### 2.4. Data Analysis

Descriptive analyses were performed using IBM SPSS Statistics 20.0 (IBM Corp., Armonk, NY, USA). We assessed distributions using the Shapiro–Wilk test and graphical methods. Continuous data were presented as mean ± standard deviation or median [25th–75th percentile], and categorical data as number (%). Because matching was implemented at the group level rather than through fixed patient–control pairs, all patient–control analyses treated the groups as independent. After the three patient withdrawals, baseline comparability of the retained groups was reassessed descriptively and by comparing age with an independent-samples *t* test, sex with Pearson’s chi-square test, and developmental age-category distributions with the Fisher–Freeman–Halton exact test. Pre- and post-transfusion hemoglobin values within the patient group were assessed using a paired *t* test, and Cohen’s d_z was calculated.

The Mann–Whitney U test was used for patient–control comparisons of linear sleep-diary and questionnaire variables; effects were reported using rank-biserial correlation and the Hodges–Lehmann difference (95% CI). These were independent-group comparisons and did not use paired or matched-set analyses. Paired questionnaire changes were examined within participants using the Pratt–Wilcoxon test, and the group × time effect was examined by comparing individual change scores between the two independent groups. Categorical variables were assessed using the chi-square or Fisher exact test, and within-group classification changes were assessed using the exact McNemar test; odds ratios (95% CIs) were reported. Three primary outcome domains were defined: (1) sleep-diary measures, including sleep regularity (within-participant standard deviation of sleep midpoint), daytime nap frequency and duration, sleep-duration variability, and nocturnal awakening measures; (2) continuous CSHQ total and subscale scores; and (3) the continuous modified PSQI global score. Analyses of the modified PSQI ≥ 5 and CSHQ ≥ 41 classifications, individual questionnaire items (including nocturnal heat sensation and OSA-related items), and all laboratory/MRI associations were exploratory. Statistical significance was not used to determine whether an outcome belonged to a primary or exploratory category.

For descriptive assessment of developmental comparability, age was categorized as 3–5, 6–12, and 13–18 years, and the distribution was compared between groups using the Fisher–Freeman–Halton exact test. These strata were not used for outcome analyses because the small cell sizes, particularly in the youngest category, precluded reliable subgroup inference.

Time variables were compared using the watson_u2_test function of the PyCircStat2 package (version 0.1.15) with 50,000 label permutations, assuming circularity. We examined linear sleep outcomes with laboratory/MR measurements using Spearman correlations, and analyzed time variables using circular–linear correlation with 9999 permutations. Permutations and FDR corrections were performed using Python version 3.12.13 (Python Software Foundation, Beaverton, OR, USA; NumPy version 2.3.5; SciPy version 1.17.0). To address multiplicity, the Benjamini–Hochberg procedure was applied within the prespecified diary and questionnaire analysis families, and FDR-adjusted q values are reported for those analyses. This procedure limits the expected proportion of false discoveries within each family but does not eliminate the risk of false-positive or selectively emphasized findings when many correlated outcomes, periods, and contrasts are examined. Laboratory/MRI associations were exploratory because of the small available sample (*n* = 23–27, depending on the variable), the number of correlations examined, and the absence of multivariable adjustment; their *p* values are unadjusted and are not interpreted as confirmatory. A two-sided *p* value < 0.05 was used only as an exploratory screening criterion, whereas q < 0.05 identified multiplicity-adjusted findings in the diary and questionnaire families. Effect estimates and confidence intervals were considered alongside q values, and component-level primary-domain and exploratory results were interpreted cautiously.

## 3. Results

Because three of the 30 patients did not complete their sleep diary, we analyzed 27 children with TDT and 30 healthy controls. The patient and control groups were similar in age (12.08 ± 3.45 and 11.43 ± 3.56 years; *p* = 0.488) and gender distribution (female: 55.6% and 50.0%; *p* = 0.675). The median age at diagnosis was 0.75 [0.42–2.75] years, the age at initiation of regular transfusions was 0.83 [0.33–2.62] years, and the annual erythrocyte transfusion amount was 190 [167.5–225.0] mL/kg. 81.5% of the patients used deferasirox, and 18.5% used a combination of deferasirox and deferiprone. The median ferritin level was 1531 [991.5–2955.5] ng/mL. Abnormal values were detected in 8.3% and 25.0% of the 24 patients with cardiac and hepatic T2* measurements, respectively. Current hemoglobin level increased from 9.66 ± 0.86 g/dL to 12.58 ± 0.98 g/dL with transfusion (mean increase 2.93 ± 0.85 g/dL; t (26) =17.97; *p* < 0.001; Cohen d_z = 3.46) (Table 2).

The developmental age-category distributions were similar between groups (Fisher–Freeman–Halton exact *p* = 0.711). In the patient group, 1 (3.7%) participant was aged 3–5 years, 14 (51.9%) were aged 6–12 years, and 12 (44.4%) were aged 13–18 years; the corresponding values in the control group were 3 (10.0%), 16 (53.3%), and 11 (36.7%), respectively (Table 2).

Table 3 presents selected representative patient–control comparisons from the primary sleep-diary domain, chosen to summarize daytime napping, sleep-duration variability, weekday sleep regularity, and a representative measure of sleep fragmentation across the transfusion cycle. The complete set of sleep-diary comparisons that remained below the global FDR threshold, including additional nocturnal-awakening, nocturnal-wakefulness, weekday, weekend, and clock-time analyses, is provided in Supplementary Table S1. Because the domain contained several correlated component measures and the study was not separately powered for each component, the findings should be interpreted collectively as multiplicity-adjusted domain-level evidence rather than as independent confirmatory endpoints. Overall, patients showed greater sleep-duration variability, more daytime napping, less regular weekday sleep timing, and a higher nocturnal-awakening frequency across the complete 14-day period.

Sleep-duration variability was higher in patients during the pre-transfusion and post-transfusion weeks and across the complete 14-day period (all FDR-adjusted q ≤ 0.026). Over 14 days, the median within-participant SD of nightly sleep duration was 68.50 [47.35–75.35] minutes in patients and 38.40 [27.85–54.52] minutes in controls, corresponding to a Hodges–Lehmann difference of 23.10 min (95% CI 10.10–35.00; q = 0.008). This estimate represents a between-group difference in night-to-night variability and should not be interpreted as a 23.10-min nightly sleep deficit (Table 3).

Weekday sleep timing was less regular in patients before transfusion and across the 14-day period (q = 0.014 and q = 0.013, respectively), whereas the post-transfusion weekday comparison was not significant after FDR adjustment (q = 0.220). The pre-transfusion wake-time distribution differed between groups, but the confidence interval for the circular mean difference included zero; no weekend comparison remained significant after FDR adjustment (Supplementary Table S1).

Within the primary questionnaire domains, the continuous modified PSQI global score was higher in patients than controls before transfusion (5.15 ± 2.78 vs. 2.57 ± 1.98; *p* < 0.001; q < 0.001). The continuous CSHQ total, sleep-behavior, and daytime-sleepiness scores were also higher in patients (all q < 0.001), whereas the CSHQ nocturnal-awakening subscale did not differ. In exploratory threshold analyses, 55.6% of patients and 13.3% of controls met the modified PSQI ≥ 5 threshold (OR = 8.12; 95% CI 2.22–29.75; *p* < 0.001; q < 0.001), and 85.2% versus 36.7% had CSHQ ≥ 41 (OR = 9.93; 95% CI 2.72–36.29; *p* < 0.001; q < 0.001).

At the post-transfusion assessment, the primary continuous modified PSQI global score no longer differed between patients and controls (*p* = 0.214; q = 0.256). Within the primary CSHQ domain, total and sleep-behavior scores remained higher in patients, whereas daytime sleepiness did not differ; the CSHQ nocturnal-awakening subscale was lower in patients. In exploratory threshold analyses, CSHQ ≥41 remained more frequent in patients (OR = 4.94; 95% CI 1.58–15.38; *p* = 0.005; q = 0.009), whereas the modified PSQI ≥5 classification did not differ (*p* = 1.000; q = 1.000) (Table 4).

Within patients, the primary continuous modified PSQI global score was lower at the post-transfusion assessment than at the pre-transfusion assessment (5.15 ± 2.78 vs. 3.15 ± 2.33; q ≤ 0.001). CSHQ total, daytime-sleepiness, and sleep-behavior scores were also lower after transfusion, whereas no corresponding multiplicity-adjusted changes were detected in controls. In the exploratory modified PSQI ≥ 5 classification, 11 patients moved below the threshold and none moved above it (q = 0.002); the CSHQ ≥ 41 classification change was not statistically significant. These temporal comparisons do not establish a causal transfusion effect. The compact between-group summary is presented in Table 4. Complete assessment-point and between-group change-score results are provided in Supplementary Table S2, and complete within-group pre-to-post analyses are provided in Supplementary Table S3.

In between-group change-score analyses within the primary questionnaire domains, differences were detected for the continuous modified PSQI global score (*p* ≤ 0.001; q ≤ 0.001), CSHQ total and daytime sleepiness (both q ≤ 0.001), sleep behavior (q = 0.012), and nocturnal awakenings (q = 0.019); however, the sleep-behavior result was not confirmed in the sensitivity analysis. The exploratory between-group difference in modified PSQI ≥5 classification change also met the FDR threshold (q ≤ 0.001) (Table 4; Supplementary Table S2). Because numerous correlated component outcomes were evaluated in a small sample, these results should be interpreted collectively within their outcome domains rather than as independent definitive effects.

Exploratory item-level analyses examined OSA-related CSHQ responses and the PSQI item concerning excessive warmth during sleep. The OSA-related questionnaire score did not differ between groups (3.67 ± 1.33 vs. 3.17 ± 0.46; U = 464.0; *p* = 0.181). Excessive warmth was reported by 77.8% (21/27) of patients and 20.0% (6/30) of controls (OR = 14.00; 95% CI 3.92–50.06; *p* ≤ 0.001), and the item-frequency score was higher in patients (2.26 ± 1.26 vs. 0.20 ± 0.41; U = 702.0; *p* ≤ 0.001). These single-item analyses were not primary outcomes, were not separately powered, and were not included in a dedicated multiplicity-controlled confirmatory family; they should therefore be considered hypothesis-generating.

In exploratory, unadjusted analyses, six associations met the nominal *p* < 0.05 threshold (Table 5). Current pre-transfusion hemoglobin correlated inversely with the pre-transfusion CSHQ daytime sleepiness score (ρ = −0.425; *p* = 0.027), and mean pre-transfusion hemoglobin over the preceding year correlated inversely with the CSHQ total score (ρ = −0.409; *p* = 0.036). Ferritin was not correlated with the pre- or post-transfusion CSHQ total, CSHQ daytime sleepiness, or modified PSQI global scores (all *p* ≥ 0.051). Complete laboratory/MRI association results, including null findings, are provided in Supplementary Table S4. Because these analyses were based on a small sample, involved multiple comparisons, used unadjusted *p* values, and were not adjusted for potential confounders, they should be regarded as hypothesis-generating rather than evidence of an independent or causal effect of hemoglobin or iron status on sleep.

## 4. Discussion

The three primary outcome domains showed a multidimensional but not uniformly consistent pattern. Within the sleep-diary domain, weekday sleep irregularity was greater in patients before transfusion and over the 14-day weekday analysis, but the post-transfusion between-group comparison was not significant; patients also had greater sleep-duration variability, more daytime naps, and more or longer reported nocturnal awakenings in selected periods. Within the questionnaire domains, the continuous modified PSQI global score and several continuous CSHQ scores were higher in patients before transfusion and decreased across the cycle, although not every measure differed at the post-transfusion assessment. These temporal findings are compatible with variation across the transfusion cycle but cannot be attributed specifically to transfusion or the rise in hemoglobin because assessment time was not randomized and other time-varying influences were not controlled. Moreover, sleep-diary measures were not verified by actigraphy.

The multiplicity-adjusted primary domains included correlated component outcomes and repeated contrasts. Greater night-to-night sleep-duration variability, reported awakenings, daytime napping, and differences in CSHQ scores should therefore be interpreted as convergent domain-level patterns rather than independent effects. Because the study was not powered for every component, replication in a larger cohort with a prospectively specified endpoint hierarchy is needed.

The magnitude and clinical meaning of the sleep-duration variability difference require caution. Over 14 days, the median within-participant standard deviation was 68.50 min in patients and 38.40 min in controls, with a Hodges–Lehmann difference of 23.10 min (95% CI 10.10–35.00; rank-biserial correlation = 0.536). This represents greater night-to-night dispersion around each child’s mean, not a 23-min nightly sleep deficit. A less predictable sequence of shorter and longer nights may complicate stable school-day wake times, alertness, and family routines even when mean sleep duration is similar. Greater variability has been associated with academic and cardiometabolic outcomes in broader pediatric populations [4,5], but these observations do not establish that the 20–25-min difference found here is clinically important. No validated clinical cut-off or minimal clinically important difference exists for diary-derived sleep-duration variability in children with TDT, the confidence interval was wide, and functional consequences were not measured. The concurrent frequency-matched control group provides an empirical reference for the magnitude and direction of the difference, but cannot substitute for a clinical threshold. Future studies should combine actigraphy and diaries with school functioning, fatigue, mood, and quality-of-life outcomes to define patient-relevant thresholds.

Although the CSHQ-derived OSA symptom score was numerically higher in patients, the difference was not statistically significant. Pediatric thalassemia findings are heterogeneous: Tarasiuk et al. reported fragmentation and periodic limb movements without OSA, whereas Sritippayawan et al. reported an OSA prevalence of approximately 8% in severe β-thalassemia [6,28]. Differences in phenotype, upper-airway anatomy, and measurement may contribute. Because questionnaire scores do not establish an OSA diagnosis, our data cannot support age-group comparisons; children with suggestive symptoms require clinical evaluation and diagnostic confirmation.

The higher frequency of reported nocturnal heat sensation was an exploratory single-item finding that was neither separately powered nor included in a dedicated multiplicity-controlled confirmatory family. Room temperature, sweating, infection, endocrine disorders, puberty, and medication effects were not assessed; therefore, this item cannot establish altered thermoregulation or autonomic dysfunction. The finding remains hypothesis-generating and requires replication with prespecified objective measures.

The inverse correlations of pre-transfusion hemoglobin with daytime sleepiness and total sleep-problem scores are hypothesis-generating and are consistent with previous reports of hemoglobin-related physical, psychosocial, or sleep outcomes [29,30,31,32,33,34,35,36,37,38]. However, these small, unadjusted analyses could not account for age, disease severity, chelation, comorbidity, or other confounders. They do not demonstrate that low hemoglobin causes sleep disturbance or that a longer transfusion interval worsens sleep; adequately powered prospective studies are required before sleep outcomes can inform transfusion targets or timing.

The relationship between iron burden and sleep is complex. Some studies report associations between ferritin and poor sleep or insomnia, whereas others do not [9,10]. In our small exploratory analysis, ferritin was not correlated with most CSHQ or modified PSQI scores, and the observed liver T2* associations require independent confirmation. These findings neither exclude an effect of organ iron nor establish a link between iron status and sleep, because ferritin is influenced by inflammation, infection, liver disease, and chelation and because organ-level measures were available for only a subset. Accordingly, no recommendation regarding transfusion timing, hemoglobin targets, or chelation practice can be derived from these data. Sleep measures may be useful as complementary outcomes in future studies, alongside hematologic, growth, organ-iron, and quality-of-life measures.

Taken together, the multiplicity-adjusted findings support multidimensional sleep assessment rather than reliance on mean duration alone. Sleep diaries may assist individualized history taking by making reported timing, variability, awakenings, and naps visible. However, the component-level and exploratory findings do not justify new screening or treatment protocols and require replication in larger, prospectively specified multicenter studies.

The study’s strengths include the inclusion of a healthy control group frequency-matched at recruitment on age, sex, and school-attendance category; repeated assessments of children throughout a transfusion cycle; exclusion of the day of hospital admission and the following day due to the potential for creating a mandatory sleep debt; and interpretation of 14-day data using validated questionnaires. Examining inter-day variability indicators such as total sleep duration standard deviation and sleep mid-time standard deviation, going beyond mean values, expands upon the literature, which is often based on cross-sectional questionnaires or single-night measurements. Comparing pre- and post-transfusion weeks in the same individual reduced the impact of inter-individual differences and made the cycle pattern more distinct.

This study has several limitations. Its single-center design and final sample of 27 patients limited precision, generalizability, age-stratified analyses, and the ability to detect anything other than relatively large effects. The wide age range (3–18 years) may also have amplified developmental differences in sleep and questionnaire comprehension.

Most importantly, sleep continuity and timing were assessed entirely using sleep diaries and questionnaires; no actigraphy was obtained. Consequently, the diary-derived nocturnal-awakening, sleep-duration-variability, and sleep-midpoint-variability measures should not be interpreted as objective measurements of sleep fragmentation or sleep–wake timing irregularity. Participant and parent reports are susceptible to recall and reporting error and may fail to detect brief awakenings or subtle changes in sleep onset and offset. Concurrent actigraphy would have strengthened the study by providing objective, multi-night estimates of sleep–wake timing, variability, and continuity and by permitting triangulation with the reported measures. Future studies should therefore combine diaries and questionnaires with actigraphy across the monitoring period.

The PSQI was originally developed as a one-month self-report instrument, whereas we used a seven-day recall period and a joint parent–child reporting procedure. Although pediatric PSQI studies include children as young as 3 years and a score of ≥5 has ROC-based support in a specific 6–18-year-old clinical population [23], the ≥5 threshold has not been validated across the full age range or modified administration studied here. Separate parent- and child-specific scores were not collected; therefore, informant effects and age-related reporting differences could not be quantified. Accordingly, the modified PSQI ≥5 and CSHQ ≥41 classifications, OSA-related items, nocturnal heat-sensation item, and laboratory/MRI associations were treated as exploratory. Numerous correlated outcomes, periods, and contrasts were examined. Although Benjamini–Hochberg FDR control was applied within the diary and questionnaire families, multiplicity correction reduces but does not eliminate the possibility of false-positive or selectively emphasized findings, particularly in a small sample. The study was not separately powered for every component measure within the primary domains or for exploratory endpoints, and the laboratory/MRI analyses used unadjusted *p* values without multivariable adjustment. Component-level primary-domain findings should be interpreted collectively, and exploratory findings should be regarded as hypothesis-generating and require independent confirmation. Finally, the observational pre–post design cannot separate the effect of transfusion or increased hemoglobin from time within the cycle, expectancy, daily routines, or other unmeasured time-varying factors.

Questionnaire administration mode differed systematically between assessments: the pre-transfusion assessment was conducted in person, whereas the post-transfusion assessment was conducted by telephone. Differences in rapport, privacy, item clarification, and response behavior across modes may therefore have contributed to the observed pre–post score changes. Because mode and assessment time were completely coupled and were not randomized or counterbalanced, a mode effect cannot be separated from temporal variation across the transfusion cycle. The concurrent control group does not eliminate this within-participant source of measurement bias. Future repeated-measures studies should use the same administration mode at all time points or randomize and counterbalance administration mode and formally assess mode equivalence.

The broad developmental age range remains an important limitation. Although controls were frequency-matched to patients at recruitment and the final groups had similar mean ages and distributions across the 3–5-, 6–12-, and 13–18-year categories, these measures reduce but do not eliminate age-related heterogeneity. Developmental stage can influence habitual sleep duration, daytime napping, bedtime and wake-time preferences, night-to-night variability, and the relative contribution of parent versus child reporting. These differences could affect diary and questionnaire outcomes independently of TDT and could also modify the magnitude of between-group or across-cycle differences. Because three patients withdrew after recruitment and no post-withdrawal rematching was performed, exact preservation of the original frequency-matching balance cannot be assumed; residual imbalance in matching variables remains possible. The sample was inadequate for reliable age-stratified inference: only one patient and three controls were aged 3–5 years, and further stratification would have produced imprecise, unstable estimates and additional multiple comparisons. We therefore presented the developmental strata descriptively rather than performing underpowered subgroup outcome analyses. The study was also not powered to test age-by-group or age-by-transfusion interactions. Larger multicenter cohorts should prespecify developmentally appropriate strata, ensure adequate representation within each stratum, and examine age as a potential effect modifier using stratified or interaction models.

## 5. Conclusions

This study indicates that reported sleep problems in children with TDT are multidimensional and vary across the transfusion cycle. Questionnaire-reported sleep problems and daytime sleepiness were lower at the post-transfusion assessment, while diary-derived nocturnal fragmentation and sleep-duration variability persisted; these temporal observations do not establish a causal benefit of transfusion or higher hemoglobin. The excess of reported nocturnal heat sensation also requires confirmation. Future multicenter studies should combine sleep diaries and questionnaires with actigraphy over longer monitoring periods, together with continuous temperature and autonomic measurements and prespecified analyses of hemoglobin and transfusion timing. Sleep variability, regularity, nocturnal integrity, and daytime function merit evaluation as complementary outcomes in such studies, but the present findings should not be used to determine transfusion intervals or hemoglobin targets.

## Figures and Tables

**Table 1:** Definitions, calculation methods, and interpretation of sleep diary-derived parameters.

Parameter	Definition/Calculation	Unit and Interpretation
**Daytime napping**	Number of naps and total duration of daytime sleep recorded in the sleep diary.	Count and min
**Sleep onset and final wake time**	Clock time of sleep onset and final morning awakening.	Clock time; circular variable
**Sleep onset latency (SOL)**	Interval between attempting to fall asleep and sleep onset.	min
**Nocturnal awakenings and wake after sleep onset (WASO)**	Number of awakenings after sleep onset and total time spent awake.	Count and min
**Total sleep time (TST)**	Time in bed (TIB) − [SOL + WASO].	min
**Sleep efficiency**	Sleep maintenance efficiency = TST/(TST + WASO) × 100 Overall sleep efficiency = TST/TIB × 100	%; higher values indicate greater efficiency
**Sleep midpoint**	Sleep onset + [(final wake time − sleep onset)/2]; clock times crossing midnight were converted to a continuous time scale.	Clock time; circadian timing
**Sleep variability**	Within-participant standard deviation of nightly TST values.	min; higher values indicate greater variability
**Sleep regularity**	Within-participant standard deviation of nightly sleep midpoints.	min; higher values indicate lower regularity
**Social jetlag**	Absolute difference between mean weekend and weekday sleep midpoints.	h/min; absolute timing difference

Abbreviations: h = hours; min = minutes; SOL = sleep onset latency; TIB = time in bed; TST = total sleep time; WASO = wake after sleep onset.

**Table 2:** Sociodemographic characteristics of the study groups and clinical, laboratory, and magnetic resonance imaging findings in the patient group.

Variable	Patient Group (*n* = 27)	Control Group (*n* = 30)	*p* Value
**A. Sociodemographic characteristics**			
Age, years; mean ± SD; median [IQR]	12.08 ± 3.45; 12.25 [10.04–14.88]	11.43 ± 3.56; 12.50 [7.88–13.33]	0.488
**Age category, years, *n* (%)**			0.711
3–5	1 (3.7)	3 (10.0)	
6–12	14 (51.9)	16 (53.3)	
13–18	12 (44.4)	11 (36.7)	
**Sex, *n* (%)**			0.675
Female	15 (55.6)	15 (50.0)	
Male	12 (44.4)	15 (50.0)	
**B. Clinical characteristics**			
Age at thalassemia diagnosis, years; mean ± SD; median [IQR]	1.49 ± 1.50; 0.75 [0.42–2.75]	—	—
Age at initiation of transfusion, years; mean ± SD; median [IQR]	2.13 ± 2.77; 0.83 [0.33–2.62]	—	—
Annual packed red blood cell transfusion volume, mL/kg; mean ± SD; median [IQR]	195.2 ± 34.2; 190.0 [167.5–225.0]	—	—
Hepatomegaly, *n* (%)	3 (11.1)	—	—
Splenectomy, *n* (%)	1 (3.7)	—	—
Chelation: deferasirox monotherapy, *n* (%)	22 (81.5)	—	—
Chelation: combined DFX + DFP therapy, *n* (%)	5 (18.5)	—	—
Abnormal cardiac T2* MRI, *n/N* (%)	2/24 (8.3)	—	—
Abnormal hepatic T2* MRI, *n/N* (%)	6/24 (25.0)	—	—
**C. Laboratory and MRI findings**			
Mean pre-transfusion Hb during the previous year, g/dL	9.69 ± 0.46; 9.70 [9.35–9.90]; 8.80–11.00	—	—
Current pre-transfusion Hb, g/dL	9.66 ± 0.86; 9.90 [9.15–10.10]; 7.10–11.20	—	—
Post-transfusion Hb, g/dL	12.58 ± 0.98; 12.50 [12.10–13.00]; 9.70–14.80	—	—
Increase in Hb (post-transfusion − pre-transfusion; calculated directly), g/dL	2.93 ± 0.85; 3.00 [2.40–3.45]; 0.70–4.90	—	<0.001 *
Ferritin, ng/mL	2290.6 ± 1915.8; 1531.0 [991.5–2955.5]; 632.0–7463.0	—	—
Cardiac T2* MRI, ms	36.33 ± 9.40; 38.50 [31.00–41.25]; 14.00–56.00	—	—
Hepatic T2* MRI, ms	10.51 ± 5.18; 12.50 [7.50–14.00]; 0.80–19.00	—	—

Note. Data are presented, as available, as *n*; mean ± SD; median [IQR]; range; or *n* (%). Age was compared between the patient and control groups using an independent-samples Student’s *t* test [t(55) = 0.70], and sex distribution was compared using Pearson’s chi-square test [χ^2^(1) = 0.18]; the omnibus *p* value for sex is reported in the Sex heading row. Developmental age-category distributions were compared using a single Fisher–Freeman–Halton exact test. * Current pre- and post-transfusion Hb values were compared using a paired-samples *t* test [t(26) = 17.97; Cohen’s d_z = 3.46]. Control-group values and *p* values were not applicable to patient-specific variables. MRI measurements were available for 24 patients. DFP, deferiprone; DFX, deferasirox; Hb, hemoglobin; IQR, interquartile range; MRI, magnetic resonance imaging; SD, standard deviation.

**Table 3:** Selected representative primary sleep-diary outcomes comparing patients and controls after global false discovery rate adjustment.

Period/ Day Type	Variable	Patient Group Mean ± SD; Median [IQR]	Control Group Mean ± SD; Median [IQR]	HL Shift (95% CI)	Effect Size (Rank-Biserial r)	*p* Value	FDR-Adjusted q
Pre-transfusion							
Weekdays	Sleep regularity (SD of sleep midpoint), min	19.90 ± 11.25; 16.30 [12.00–22.80]	12.42 ± 8.44; 10.15 [8.38–12.78]	+5.85 (2.60–10.90)	0.477	0.002	0.014
All days	Daytime nap frequency, naps/day	0.11 ± 0.17; 0.00 [0.00–0.14]	0.02 ± 0.09; 0.00 [0.00–0.00]	0.00 (0.00–0.14)	0.300	0.007	0.026
All days	Mean daytime sleep duration, min	7.83 ± 13.30; 0.00 [0.00–9.70]	1.27 ± 5.92; 0.00 [0.00–0.00]	0.00 (0.00–8.60)	0.307	0.005	0.026
All days	Sleep duration variability, min	67.73 ± 36.23; 65.60 [43.15–78.85]	43.80 ± 27.56; 35.80 [24.90–56.62]	+22.60 (8.00–37.95)	0.427	0.006	0.026
Post-transfusion							
All days	Sleep duration variability, min	66.43 ± 34.20; 60.00 [50.55–73.25]	43.84 ± 22.54; 39.35 [26.30–52.62]	+19.85 (7.10–33.00)	0.437	0.005	0.026
14-day period							
Weekdays	Sleep regularity (SD of sleep midpoint), min	22.17 ± 8.94; 18.90 [15.85–26.40]	15.65 ± 8.25; 13.00 [10.25–17.73]	+6.45 (2.80–9.90)	0.481	0.002	0.013
All days	Daytime nap frequency, naps/day	0.08 ± 0.12; 0.00 [0.00–0.14]	0.02 ± 0.07; 0.00 [0.00–0.00]	0.00 (0.00–0.07)	0.374	0.002	0.015
All days	Mean daytime sleep duration, min	6.09 ± 8.86; 1.45 [0.00–11.15]	1.10 ± 4.01; 0.00 [0.00–0.00]	0.00 (0.00–6.00)	0.400	0.001	0.012
All days	Nocturnal awakenings, events/night	0.12 ± 0.22; 0.00 [0.00–0.17]	0.00 ± 0.00; 0.00 [0.00–0.00]	0.00 (0.00–0.14)	0.370	<0.001	0.005
All days	Sleep duration variability, min	68.87 ± 30.72; 68.50 [47.35–75.35]	43.90 ± 23.10; 38.40 [27.85–54.52]	+23.10 (10.10–35.00)	0.536	<0.001	0.008

Note. Table 3 presents selected representative outcomes; the complete set of multiplicity-adjusted sleep-diary comparisons is provided in Supplementary Table S1. Data are presented as mean ± SD and median [IQR]. Mean ± SD is included as a descriptive statistic; inferential analyses were rank-based. Sleep-duration variability was defined as the within-participant SD of total sleep time, and sleep regularity as the within-participant SD of sleep midpoint; higher values indicate greater duration variability and less regular sleep timing, respectively. The Hodges–Lehmann (HL) shift and its 95% CI are reported in the units specified in the Variable column; the rank-biserial correlation summarizes effect magnitude. Groups were compared using the Mann–Whitney U test. Test statistics are omitted from the condensed main table and are reported with the complete period-specific results in Supplementary Table S1. CI, confidence interval; FDR, false discovery rate; IQR, interquartile range; SD, standard deviation. A global Benjamini–Hochberg FDR-adjusted q value <0.05 was considered statistically significant. Because of zero-value clustering in nocturnal-awakening outcomes, identical medians may coexist with differences in rank distributions. To minimize redundancy, closely related fragmentation outcomes with identical or near-identical effect estimates were retained in Supplementary Table S1.

**Table 4:** Compact summary of questionnaire outcomes before and after transfusion and of between-group differences in pre-to-post change.

Outcome	Pre-Transfusion Comparison	Post-Transfusion Comparison	Pre-to-Post Change Comparison
CSHQ total score	P: 52 [47–56.5] C: 40 [38–44] r_rb = +0.726; q ≤ 0.001	P: 48 [41–53.5] C: 40 [38–44] r_rb = +0.486; q = 0.004	ΔP: 2 [0–7] ΔC: 0 [0–0] q ≤ 0.001
CSHQ score ≥ 41: clinically significant sleep problems	P: 23/27 (85.2%) C: 11/30 (36.7%) OR = 9.93; 95% CI 2.72–36.29; q ≤ 0.001	P: 20/27 (74.1%) C: 11/30 (36.7%) OR = 4.94; 95% CI 1.58–15.38; q = 0.009	P: 3/24/0 C: 0/30/0 ΔΔ = +11.1 pp; q = 0.099
CSHQ bedtime resistance	P: 12 [9–16] C: 10 [10–12] r_rb = +0.210; q = 0.173	P: 12 [9–16] C: 10 [10–12] r_rb = +0.144; q = 0.347	ΔP: 0 [0–0] ΔC: 0 [0–0] q = 0.056
CSHQ sleep behavior problems	P: 22 [19.5–23.5] C: 17 [16–17] r_rb = +0.832; q ≤ 0.001	P: 20 [18.5–23] C: 17 [16–17] r_rb = +0.789; q ≤ 0.001	ΔP: 0 [0–0] ΔC: 0 [0–0] q = 0.012
CSHQ night wakings	P: 2 [2–2.5] C: 3 [2–3] r_rb = −0.225; q = 0.126	P: 2 [2–2] C: 3 [2–3] r_rb = −0.356; q = 0.011	ΔP: 0 [0–0] ΔC: 0 [0–0] q = 0.019
CSHQ daytime sleepiness	P: 16 [12–17.5] C: 11 [8–12] r_rb = +0.663; q ≤ 0.001	P: 11 [9–14.5] C: 11 [8–12] r_rb = +0.209; q = 0.256	ΔP: 2 [0–6] ΔC: 0 [0–0] q ≤ 0.001
PSQI global score	P: 5 [3–7] C: 2 [1–3] r_rb = +0.569; q ≤ 0.001	P: 2 [2–4] C: 2 [1–3] r_rb = +0.188; q = 0.256	ΔP: 1 [0.5–3.5] ΔC: 0 [0–0] q ≤ 0.001
Modified PSQI score ≥ 5: exploratory threshold	P: 15/27 (55.6%) C: 4/30 (13.3%) OR = 8.12; 95% CI 2.22–29.75; q ≤ 0.001	P: 4/27 (14.8%) C: 4/30 (13.3%) OR = 1.13; 95% CI 0.25–5.04; q = 1.000	P: 11/16/0 C: 0/30/0 ΔΔ = +40.7 pp; q ≤0.001

Note. Values in the compact main table are median [IQR] for continuous outcomes and *n/N* (%) for threshold outcomes. P and C denote the patient and control groups, respectively. Δ was calculated as the pre-transfusion value minus the post-transfusion value; positive continuous values indicate lower post-transfusion scores. Rank-biserial correlations (r_rb) are shown for continuous between-group comparisons at each assessment, and odds ratios with 95% confidence intervals are shown for threshold outcomes. For classification change, values are ordered as moved below/unchanged/moved above threshold, and ΔΔ denotes the between-group difference in mean classification change. Statistical interpretation was based on Benjamini–Hochberg FDR-adjusted q values. Complete assessment-point and between-group change-score results, including mean ± SD, unadjusted *p* values, test statistics, and effect estimates, are provided in Supplementary Table S2. Complete within-group pre-to-post analyses are provided in Supplementary Table S3. CI, confidence interval; CSHQ, Children’s Sleep Habits Questionnaire; FDR, false discovery rate; IQR, interquartile range; PSQI, Pittsburgh Sleep Quality Index.

**Table 5:** Selected nominal associations of laboratory and magnetic resonance imaging findings with sleep-diary and questionnaire outcomes in the patient group.

Laboratory or MRI Measure	Sleep Outcome	*n*	Coefficient	*p* Value
**Sleep diary (14-day period)**				
Hepatic T2* MRI, ms	Daytime sleep duration, min	23	ρ = −0.6059	0.002
Hepatic T2* MRI, ms	Number of daytime naps	24	ρ = −0.4805	0.019
Current pre-transfusion Hb, g/dL	Sleep midpoint, clock time	27	r_cl = 0.4910	0.035
Cardiac T2* MRI, ms	Wake time, clock time	24	r_cl = 0.4991	0.044
**Questionnaire (pre-transfusion)**				
Current pre-transfusion Hb, g/dL	CSHQ daytime sleepiness score	27	ρ = −0.4254	0.027
Mean pre-transfusion Hb during the previous year, g/dL	CSHQ total score	27	ρ = −0.4094	0.036

Note. Only associations with nominal unadjusted *p* < 0.05 are displayed in the main table to improve readability; all reported laboratory/MRI association analyses, including null findings, are presented in Supplementary Table S4. ρ denotes Spearman’s rank correlation coefficient, and r_cl denotes the unsigned rank-based circular–linear correlation coefficient. No multiplicity adjustment was applied. These analyses were not separately powered and should be interpreted as exploratory and hypothesis-generating. CSHQ, Children’s Sleep Habits Questionnaire; Hb, hemoglobin; MRI, magnetic resonance imaging.

## Data Availability

The original contributions presented in this study are included in the article. Further enquiries can be directed to the corresponding author.

## Supplementary Materials

The following supporting information can be downloaded at: https://www.mdpi.com/article/10.3390/children13101339/s1, Supplementary Table S1: Complete multiplicity-adjusted sleep-diary comparisons between patients and controls, including outcomes omitted from the condensed main Table 3; Supplementary Table S2: Complete between-group questionnaire analyses at the pre-transfusion and post-transfusion assessments and for pre-to-post change scores; Supplementary Table S3: Complete within-group absolute changes from pre-transfusion to post-transfusion in questionnaire scores and clinical classifications; Supplementary Table S4: Complete exploratory associations of laboratory and magnetic resonance imaging findings with sleep-diary and questionnaire outcomes in the patient group.
