# Supplementary material for: Sleep Patterns Across the Transfusion Cycle in Children with Transfusion-Dependent β-Thalassemia: A 14-Day Prospective Controlled Study Using Sleep Diaries and Repeated Questionnaires
Source: Children (Basel). 2026 Oct 2;13(10):1339. doi: 10.3390/children13101339 (PMC13703031; doi:10.3390/children13101339)
Supplement: Supplementary file 1 [file children-13-01339-s001.zip › Supplementary Table S1.pdf]

Supplementary Table S1. Complete multiplicity-adjusted sleep-diary comparisons between patients and controls, including outcomes omitted from the condensed main Table 3.

**Period / day type**	**Variable**	**Patient group Mean ± SD; median [IQR]**	**Control group Mean ± SD; median [IQR]**	Effect estimate (95% CI) HL shift / Δcircular	Effect size (rank-biserial r)	Test statistic (U / U²)	**p value**	**FDR-adjusted q**
**Pre-transfusion**								
Weekdays	Nocturnal awakenings, events/night	0.13 ± 0.27; 0.00 [0.00–0.00]	0.00 ± 0.00; 0.00 [0.00–0.00]	0.00 (0.00–0.00)	0.222	495.0	0.007	0.026
Weekdays	Mean nocturnal wakefulness duration, min	2.62 ± 6.44; 0.00 [0.00–0.00]	0.00 ± 0.00; 0.00 [0.00–0.00]	0.00 (0.00–0.00)	0.222	495.0	0.007	0.026
Weekdays	Sleep duration variability, min	45.09 ± 24.88; 38.70 [28.95–52.75]	25.53 ± 15.60; 22.80 [16.92–31.80]	+16.00 (7.60–26.30)	0.530	619.5	<0.001	0.008
Weekdays	Sleep regularity (SD of sleep midpoint), min	19.90 ± 11.25; 16.30 [12.00–22.80]	12.42 ± 8.44; 10.15 [8.38–12.78]	+5.85 (2.60–10.90)	0.477	598.0	0.002	0.014
All days	Daytime nap frequency, naps/day	0.11 ± 0.17; 0.00 [0.00–0.14]	0.02 ± 0.09; 0.00 [0.00–0.00]	0.00 (0.00–0.14)	0.300	526.5	0.007	0.026
All days	Mean daytime sleep duration, min	7.83 ± 13.30; 0.00 [0.00–9.70]	1.27 ± 5.92; 0.00 [0.00–0.00]	0.00 (0.00–8.60)	0.307	529.5	0.005	0.026
All days	Mean wake time, clock time	08:18 (circular SD 56.5 min)	08:06 (circular SD 56.5 min)	+12.0 min (−18.4–40.3)	—	0.309	0.004	0.021
All days	Nocturnal awakenings, events/night	0.12 ± 0.25; 0.00 [0.00–0.00]	0.00 ± 0.00; 0.00 [0.00–0.00]	0.00 (0.00–0.00)	0.222	495.0	0.007	0.026
All days	Mean nocturnal wakefulness duration, min	2.33 ± 5.56; 0.00 [0.00–0.00]	0.00 ± 0.00; 0.00 [0.00–0.00]	0.00 (0.00–0.00)	0.222	495.0	0.007	0.026
All days	Sleep duration variability, min	67.73 ± 36.23; 65.60 [43.15–78.85]	43.80 ± 27.56; 35.80 [24.90–56.62]	+22.60 (8.00–37.95)	0.427	578.0	0.006	0.026
**Post-transfusion**								
Weekdays	Nocturnal awakenings, events/night	0.12 ± 0.24; 0.00 [0.00–0.20]	0.00 ± 0.00; 0.00 [0.00–0.00]	0.00 (0.00–0.00)	0.296	525.0	0.002	0.012
Weekdays	Mean nocturnal wakefulness duration, min	1.44 ± 2.71; 0.00 [0.00–1.50]	0.00 ± 0.00; 0.00 [0.00–0.00]	0.00 (0.00–0.00)	0.296	525.0	0.002	0.012
All days	Nocturnal awakenings, events/night	0.12 ± 0.24; 0.00 [0.00–0.14]	0.00 ± 0.00; 0.00 [0.00–0.00]	0.00 (0.00–0.00)	0.296	525.0	0.002	0.012
All days	Mean nocturnal wakefulness duration, min	1.41 ± 2.79; 0.00 [0.00–2.30]	0.00 ± 0.00; 0.00 [0.00–0.00]	0.00 (0.00–0.00)	0.296	525.0	0.002	0.012
All days	Sleep duration variability, min	66.43 ± 34.20; 60.00 [50.55–73.25]	43.84 ± 22.54; 39.35 [26.30–52.62]	+19.85 (7.10–33.00)	0.437	582.0	0.005	0.026

Period / day type	Variable	Patient group Mean ± SD; median [IQR]	Control group Mean ± SD; median [IQR]	Effect estimate (95% CI) HL shift / Δcircular	Effect size (rank-biserial r)	Test statistic (U / U²)	p value	FDR-adjusted q
**14-day period**								
Weekdays	Nocturnal awakenings, events/night	0.12 ± 0.22; 0.00 [0.00–0.20]	0.00 ± 0.00; 0.00 [0.00–0.00]	0.00 (0.00–0.10)	0.370	555.0	<0.001	0.005
Weekdays	Mean nocturnal wakefulness duration, min	2.02 ± 4.39; 0.00 [0.00–2.00]	0.00 ± 0.00; 0.00 [0.00–0.00]	0.00 (0.00–1.50)	0.370	555.0	<0.001	0.005
Weekdays	Sleep duration variability, min	48.37 ± 21.16; 43.10 [36.30–55.40]	30.06 ± 16.27; 30.15 [21.05–35.43]	+16.00 (8.10–25.60)	0.575	638.0	<0.001	0.005
Weekdays	Sleep regularity (SD of sleep midpoint), min	22.17 ± 8.94; 18.90 [15.85–26.40]	15.65 ± 8.25; 13.00 [10.25–17.73]	+6.45 (2.80–9.90)	0.481	600.0	0.002	0.013
Weekend days	Nocturnal awakenings, events/night	0.10 ± 0.24; 0.00 [0.00–0.00]	0.00 ± 0.00; 0.00 [0.00–0.00]	0.00 (0.00–0.00)	0.222	495.0	0.007	0.026
Weekend days	Mean nocturnal wakefulness duration, min	1.46 ± 3.94; 0.00 [0.00–0.00]	0.00 ± 0.00; 0.00 [0.00–0.00]	0.00 (0.00–0.00)	0.222	495.0	0.007	0.026
All days	Daytime nap frequency, naps/day	0.08 ± 0.12; 0.00 [0.00–0.14]	0.02 ± 0.07; 0.00 [0.00–0.00]	0.00 (0.00–0.07)	0.374	556.5	0.002	0.015
All days	Mean daytime sleep duration, min	6.09 ± 8.86; 1.45 [0.00–11.15]	1.10 ± 4.01; 0.00 [0.00–0.00]	0.00 (0.00–6.00)	0.400	546.0	0.001	0.012
All days	Nocturnal awakenings, events/night	0.12 ± 0.22; 0.00 [0.00–0.17]	0.00 ± 0.00; 0.00 [0.00–0.00]	0.00 (0.00–0.14)	0.370	555.0	<0.001	0.005
All days	Mean nocturnal wakefulness duration, min	1.86 ± 3.95; 0.00 [0.00–1.75]	0.00 ± 0.00; 0.00 [0.00–0.00]	0.00 (0.00–1.40)	0.370	555.0	<0.001	0.005
All days	Sleep duration variability, min	68.87 ± 30.72; 68.50 [47.35–75.35]	43.90 ± 23.10; 38.40 [27.85–54.52]	+23.10 (10.10–35.00)	0.536	622.0	<0.001	0.008

Note. Except for clock-time variables, data are presented as mean ± SD and median [IQR]. Mean ± SD is provided as a supplementary descriptive statistic; inferential analyses were rank based. Sleep duration variability was defined as the within-person SD of total sleep time, and sleep regularity as the within-person SD of sleep midpoint; higher values indicate greater duration variability and less regular sleep timing, respectively. Circular mean (circular SD) was used for clock-time variables. Linear variables were compared using the Mann-Whitney U test, whereas clock-time variables were compared using the Watson two-sample U^2 test with 50,000 permutations. CI, confidence interval; circular SD, circular standard deviation; FDR, false discovery rate; HL, Hodges-Lehmann patient-control location shift; IQR, interquartile range; r_rb, rank-biserial correlation; Δcircular, shortest signed circular mean difference. A global Benjamini-Hochberg FDR-adjusted q value <0.05 was considered statistically significant. Because of zero-value clustering in nocturnal awakening outcomes, identical medians may coexist with differences in rank distributions. In the effect-estimate column, HL shift denotes the Hodges–Lehmann patient–control location shift for linear outcomes, whereas Δcircular denotes the shortest signed circular mean difference for clock-time outcomes; units are specified in the Variable column, except for Δcircular, which is reported in minutes. The U/U^2 column gives the Mann–Whitney U or Watson U^2 statistic, as applicable. This table provides the complete set of multiplicity-adjusted comparisons, including closely related fragmentation outcomes omitted from the condensed main Table 3.
