# Supplementary material for: Sleep Patterns Across the Transfusion Cycle in Children with Transfusion-Dependent β-Thalassemia: A 14-Day Prospective Controlled Study Using Sleep Diaries and Repeated Questionnaires
Source: Children (Basel). 2026 Oct 2;13(10):1339. doi: 10.3390/children13101339 (PMC13703031; doi:10.3390/children13101339)
Supplement: Supplementary file 1 [file children-13-01339-s001.zip › Supplementary Table S2.pdf]

Supplementary Table S2. Complete between-group questionnaire analyses at the pre-transfusion and post-transfusion assessments and for pre-to-post change scores.

Panel A. Between-group comparisons at the pre-transfusion and post-transfusion assessments

Measure/outcome	Patient group (n = 27) Mean ± SD; median [IQR] or n (%)	Control group (n = 30) Mean ± SD; median [IQR] or n (%)	Test statistic	Effect size	p value	FDR-adjusted q
Pre-transfusion period						
CSHQ total score	51.37 ± 8.02<br>52 [47–56.5]	40.53 ± 4.31<br>40 [38–44]	U = 699	r_rb = +0.726	<0.001	<0.001
CSHQ score ≥41: clinically significant sleep problems	23/27 (85.2%)	11/30 (36.7%)	χ^2 = 13.90	OR = 9.93<br>95% CI 2.72–36.29	<0.001	<0.001
CSHQ bedtime resistance	12.70 ± 3.98<br>12 [9–16]	10.97 ± 2.27<br>10 [10–12]	U = 490	r_rb = +0.210	0.173	0.173
CSHQ sleep behavior problems	21.74 ± 3.77<br>22 [19.5–23.5]	16.80 ± 1.27<br>17 [16–17]	U = 742	r_rb = +0.832	<0.001	<0.001
CSHQ night wakings	2.48 ± 0.98<br>2 [2–2.5]	2.67 ± 0.71<br>3 [2–3]	U = 314	r_rb = −0.225	0.105	0.126
CSHQ daytime sleepiness	14.81 ± 3.70<br>16 [12–17.5]	10.10 ± 2.64<br>11 [8–12]	U = 673.5	r_rb = +0.663	<0.001	<0.001
PSQI global score	5.15 ± 2.78<br>5 [3–7]	2.57 ± 1.98<br>2 [1–3]	U = 635.5	r_rb = +0.569	<0.001	<0.001
Modified PSQI score ≥5: exploratory threshold	15/27 (55.6%)	4/30 (13.3%)	χ^2 = 11.40	OR = 8.12<br>95% CI 2.22–29.75	<0.001	<0.001
Post-transfusion period						
CSHQ total score	47.30 ± 7.64<br>48 [41–53.5]	40.87 ± 4.83<br>40 [38–44]	U = 602	r_rb = +0.486	0.001	0.004
CSHQ score ≥41: clinically significant sleep problems	20/27 (74.1%)	11/30 (36.7%)	χ^2 = 8.02	OR = 4.94<br>95% CI 1.58–15.38	0.005	0.009
CSHQ bedtime resistance	12.48 ± 4.03<br>12 [9–16]	11.00 ± 2.35<br>10 [10–12]	U = 463.5	r_rb = +0.144	0.347	0.347
CSHQ sleep behavior problems	21.00 ± 3.40<br>20 [18.5–23]	16.80 ± 1.27<br>17 [16–17]	U = 724.5	r_rb = +0.789	<0.001	<0.001
CSHQ night wakings	2.33 ± 0.92<br>2 [2–2]	2.93 ± 1.31<br>3 [2–3]	U = 261	r_rb = −0.356	0.006	0.011
CSHQ daytime sleepiness	11.63 ± 3.75<br>11 [9–14.5]	10.13 ± 2.66<br>11 [8–12]	U = 489.5	r_rb = +0.209	0.177	0.256
PSQI global score	3.15 ± 2.33<br>2 [2–4]	2.70 ± 2.29<br>2 [1–3]	U = 481	r_rb = +0.188	0.214	0.256

Measure/outcome	Patient group (n = 27) Mean ± SD; median [IQR] or n (%)	Control group (n = 30) Mean ± SD; median [IQR] or n (%)	Test statistic	Effect size	p value	FDR-adjusted q
Modified PSQI score ≥5: exploratory threshold	4/27 (14.8%)	4/30 (13.3%)	Fisher's exact	OR = 1.13<br>95% CI 0.25–5.04	1.000	1.000

Note. Continuous scores are presented as mean ± SD and median [IQR] and were compared using two-sided Mann-Whitney U tests with 200,000 label permutations. Threshold outcomes are presented as n/N (%) and were compared using Pearson's chi-square test or Fisher's exact test when the expected cell count was <5; odds ratios (ORs) are reported with 95% confidence intervals. The six continuous outcomes and the two threshold outcomes were adjusted as separate FDR families within each period using the Benjamini-Hochberg method. CI, confidence interval; CSHQ, Children's Sleep Habits Questionnaire; FDR, false discovery rate; IQR, interquartile range; PSQI, Pittsburgh Sleep Quality Index; r_rb, rank-biserial correlation; SD, standard deviation.

Panel B. Between-group comparisons of pre-to-post change scores and classification changes

Outcome	Patient group, Δ/class change	Control group, Δ/class change	U or ΔΔ	p value	FDR-adjusted q
CSHQ total score	4.07 ± 4.64<br>2 [0–7]	−0.33 ± 0.76<br>0 [0–0]	U = 672.5	<0.001	<0.001
CSHQ bedtime resistance	0.22 ± 0.64<br>0 [0–0]	−0.03 ± 0.18<br>0 [0–0]	U = 462	0.056	0.056
CSHQ sleep behavior problems	0.74 ± 1.51<br>0 [0–0]	0.00 ± 0.00<br>0 [0–0]	U = 495	0.008	0.012
CSHQ night wakings	0.15 ± 0.46<br>0 [0–0]	−0.27 ± 0.69<br>0 [0–0]	U = 498	0.016	0.019
CSHQ daytime sleepiness	3.19 ± 3.63<br>2 [0–6]	−0.03 ± 0.18<br>0 [0–0]	U = 621.5	<0.001	<0.001
PSQI global score	2.00 ± 2.00<br>1 [0.5–3.5]	−0.13 ± 0.35<br>0 [0–0]	U = 719	<0.001	<0.001
CSHQ score ≥41: clinically significant sleep problems	Below/unchanged/above threshold<br>3/24/0	Below/unchanged/above threshold<br>0/30/0	ΔΔ = +11.1 pp	0.099	0.099
Modified PSQI score ≥5: exploratory threshold	Below/unchanged/above threshold<br>11/16/0	Below/unchanged/above threshold<br>0/30/0	ΔΔ = +40.7 pp	<0.001	<0.001

Note. For continuous outcomes, Δ was calculated as the pre-transfusion value minus the post-transfusion value, and the groups were compared using the Mann-Whitney U test. For threshold outcomes, the individual classification-change score (moved below threshold = +1, unchanged = 0, moved above threshold = −1) was compared between groups using 200,000 label permutations; ΔΔ denotes the between-group difference in mean change. Multiplicity was controlled within prespecified FDR families using the Benjamini-Hochberg method. CSHQ, Children's Sleep Habits Questionnaire; FDR, false discovery rate; IQR, interquartile range; pp, percentage points; PSQI, Pittsburgh Sleep Quality Index; SD, standard deviation. Classification-change labels are descriptive of assessment timing and do not imply causation.
