# Supplementary material for: Sleep Patterns Across the Transfusion Cycle in Children with Transfusion-Dependent β-Thalassemia: A 14-Day Prospective Controlled Study Using Sleep Diaries and Repeated Questionnaires
Source: Children (Basel). 2026 Oct 2;13(10):1339. doi: 10.3390/children13101339 (PMC13703031; doi:10.3390/children13101339)
Supplement: Supplementary file 1 [file children-13-01339-s001.zip › Supplementary Table S3.pdf]

Supplementary Table S3. Complete within-group absolute changes from pre-transfusion to post-transfusion in questionnaire scores and clinical classifications.

Variable	Group	Pre-transfusion period	Post-transfusion period	Δ (pre-transfusion − post-transfusion)	Test statistic	p value	FDR-adjusted q	Effect/discordant pairs
CSHQ total score	Patient group	51.37 ± 8.02<br>52 [47–56.5]	47.30 ± 7.64<br>48 [41–53.5]	4.07 ± 4.64<br>2 [0–7]	T = 0	<0.001	<0.001	r_rb = +1.000
CSHQ total score	Control group	40.53 ± 4.31<br>40 [38–44]	40.87 ± 4.83<br>40 [38–44]	−0.33 ± 0.76<br>0 [0–0]	T = 0	0.062	0.250	r_rb = −1.000
CSHQ bedtime resistance	Patient group	12.70 ± 3.98<br>12 [9–16]	12.48 ± 4.03<br>12 [9–16]	0.22 ± 0.64<br>0 [0–0]	T = 0	0.250	0.250	r_rb = +1.000
CSHQ bedtime resistance	Control group	10.97 ± 2.27<br>10 [10–12]	11.00 ± 2.35<br>10 [10–12]	−0.03 ± 0.18<br>0 [0–0]	T = 0	1.000	1.000	r_rb = −1.000
CSHQ sleep behavior problems	Patient group	21.74 ± 3.77<br>22 [19.5–23.5]	21.00 ± 3.40<br>20 [18.5–23]	0.74 ± 1.51<br>0 [0–0]	T = 0	0.031	0.047	r_rb = +1.000
CSHQ sleep behavior problems	Control group	16.80 ± 1.27<br>17 [16–17]	16.80 ± 1.27<br>17 [16–17]	0.00 ± 0.00<br>0 [0–0]	T = 0	1.000	1.000	r_rb = 0.000
CSHQ night wakings	Patient group	2.48 ± 0.98<br>2 [2–2.5]	2.33 ± 0.92<br>2 [2–2]	0.15 ± 0.46<br>0 [0–0]	T = 0	0.250	0.250	r_rb = +1.000
CSHQ night wakings	Control group	2.67 ± 0.71<br>3 [2–3]	2.93 ± 1.31<br>3 [2–3]	−0.27 ± 0.69<br>0 [0–0]	T = 0	0.125	0.250	r_rb = −1.000
CSHQ daytime sleepiness	Patient group	14.81 ± 3.70<br>16 [12–17.5]	11.63 ± 3.75<br>11 [9–14.5]	3.19 ± 3.63<br>2 [0–6]	T = 0	<0.001	<0.001	r_rb = +1.000
CSHQ daytime sleepiness	Control group	10.10 ± 2.64<br>11 [8–12]	10.13 ± 2.66<br>11 [8–12]	−0.03 ± 0.18<br>0 [0–0]	T = 0	1.000	1.000	r_rb = −1.000
PSQI global score	Patient group	5.15 ± 2.78<br>5 [3–7]	3.15 ± 2.33<br>2 [2–4]	2.00 ± 2.00<br>1 [0.5–3.5]	T = 0	<0.001	<0.001	r_rb = +1.000
PSQI global score	Control group	2.57 ± 1.98<br>2 [1–3]	2.70 ± 2.29<br>2 [1–3]	−0.13 ± 0.35<br>0 [0–0]	T = 0	0.125	0.250	r_rb = −1.000
CSHQ score ≥41: clinically significant sleep problems	Patient group	23/27 (85.2%)	20/27 (74.1%)	+11.1 percentage points<br>Below/above threshold = 3/0	Exact McNemar	0.250	0.250	Discordant pairs = 3

Variable	Group	Pre-transfusion period	Post-transfusion period	Δ (pre-transfusion − post-transfusion)	Test statistic	p value	FDR-adjusted q	Effect/discordant pairs
CSHQ score ≥41: clinically significant sleep problems	Control group	11/30 (36.7%)	11/30 (36.7%)	0.0 percentage points Below/above threshold = 0/0	Exact McNemar	1.000	1.000	Discordant pairs = 0
Modified PSQI score ≥5: exploratory threshold	Patient group	15/27 (55.6%)	4/27 (14.8%)	+40.7 percentage points Below/above threshold = 11/0	Exact McNemar	<0.001	0.002	Discordant pairs = 11
Modified PSQI score ≥5: exploratory threshold	Control group	4/30 (13.3%)	4/30 (13.3%)	0.0 percentage points Below/above threshold = 0/0	Exact McNemar	1.000	1.000	Discordant pairs = 0

Note. For continuous outcomes, data are presented as mean ± SD and median [IQR]. Δ was calculated as the pre-transfusion value minus the post-transfusion value; a positive change in a continuous score indicates a lower score after transfusion, whereas a positive percentage-point change indicates a decrease in problem prevalence. Continuous outcomes were evaluated using exact or permutation-based Wilcoxon signed-rank tests with zero differences handled using the Pratt method; binary classifications were evaluated using the exact McNemar test. Multiplicity was controlled within prespecified FDR families using the Benjamini-Hochberg method. Moved below threshold indicates that the classification was present at the pre-transfusion assessment but absent at the post-transfusion assessment; moved above threshold indicates the reverse. These labels describe temporal classification changes and do not imply a causal transfusion effect. CSHQ, Children's Sleep Habits Questionnaire; FDR, false discovery rate; IQR, interquartile range; PSQI, Pittsburgh Sleep Quality Index; r_rb, rank-biserial correlation; SD, standard deviation. The Δ column therefore reports the absolute point change for each continuous questionnaire score.
