# Supplementary material for: Sleep Patterns Across the Transfusion Cycle in Children with Transfusion-Dependent β-Thalassemia: A 14-Day Prospective Controlled Study Using Sleep Diaries and Repeated Questionnaires
Source: Children (Basel). 2026 Oct 2;13(10):1339. doi: 10.3390/children13101339 (PMC13703031; doi:10.3390/children13101339)
Supplement: Supplementary file 1 [file children-13-01339-s001.zip › Supplementary Table S4.pdf]

Supplementary Table S4. Complete exploratory associations of laboratory and magnetic resonance imaging findings with sleep-diary and questionnaire outcomes in the patient group.

Laboratory or MRI measure	Sleep outcome	n	Coefficient	p value
**Sleep diary (14-day period)**				
Hepatic T2* MRI, ms	Daytime sleep duration, min	23	ρ = −0.6059	0.002
Hepatic T2* MRI, ms	Number of daytime naps	24	ρ = −0.4805	0.019
Current pre-transfusion Hb, g/dL	Sleep midpoint, clock time	27	r_cl = 0.4910	0.035
Cardiac T2* MRI, ms	Wake time, clock time	24	r_cl = 0.4991	0.044
**Questionnaire (pre-transfusion)**				
Current pre-transfusion Hb, g/dL	CSHQ daytime sleepiness score	27	ρ = −0.4254	0.027
Mean pre-transfusion Hb during the previous year, g/dL	CSHQ total score	27	ρ = −0.4094	0.036
Ferritin, ng/mL	CSHQ total score	27	ρ = 0.1783	0.373
Ferritin, ng/mL	Modified PSQI global score	27	ρ = 0.3920	0.053
**Questionnaire (post-transfusion)**				
Ferritin, ng/mL	CSHQ daytime sleepiness score	27	ρ = −0.4060	0.051
Ferritin, ng/mL	CSHQ total score	27	ρ = −0.0755	0.708
Ferritin, ng/mL	Modified PSQI global score	27	ρ = −0.0369	0.855

Note. ρ denotes Spearman's rank correlation coefficient, and r_cl denotes the unsigned rank-based circular–linear correlation coefficient. Reported p values are unadjusted. These analyses were not separately powered, involved multiple correlations, and should be interpreted as exploratory and hypothesis-generating. CSHQ, Children's Sleep Habits Questionnaire; Hb, hemoglobin; MRI, magnetic resonance imaging; PSQI, Pittsburgh Sleep Quality Index.
